# Validation of the Portuguese Version of Impulsive–Premeditated Aggression Scale in an Inmate Population

**DOI:** 10.3389/fpsyt.2018.00010

**Published:** 2018-02-05

**Authors:** Jacinto Costa Azevedo, José Luís Pais-Ribeiro, Rui Coelho, Margarida Figueiredo-Braga

**Affiliations:** ^1^Department of Neuroscience and Mental Health, Faculty of Medicine, University of Porto, Porto, Portugal; ^2^I3S – Instituto de Investigação e Inovação em Saúde, Porto, Portugal; ^3^Faculty of Psychology and Educational Sciences, University of Porto, Porto, Portugal

**Keywords:** aggression, antisocial personality disorder, psychopathy, impulsivity, impulsive–premeditated aggression scale, Barratt Impulsiveness scale, 11th version, Psychopathic Checklist Revised

## Abstract

Aggression is one of the core symptoms of antisocial personality disorder (ASPD) with therapeutic and prognostic relevance. ASPD is highly prevalent among inmates, being responsible for adverse events and elevated direct and indirect economic costs for the criminal justice system. The Impulsive/Premeditated Aggression Scale (IPAS) is a self-report instrument that characterizes aggression as either predominately impulsive or premeditated. This study aims to determine the validity and reliability of the IPAS in a sample of Portuguese inmates. A total of 240 inmates were included in the study. A principal component factor analysis was performed so as to obtain the construct validity of the IPAS impulsive aggression (IA) and premeditated aggression (PM) subscales; internal consistency was determined by Cronbach’s alpha coefficient; convergent and divergent validity of the subscales were determined analyzing correlations with the Barratt Impulsiveness scale, 11th version (BIS-11), and the Psychopathic Checklist Revised (PCL-R). The rotated matrix with two factors accounted for 49.9% of total variance. IA subscale had 11 items and PM subscale had 10 items. The IA and PM subscales had a good Cronbach’s alpha values of 0.89 and 0.88, respectively. The IA subscale is correlated with BIS-11 attentional, motor, and non-planning impulsiveness dimensions (*p* < 0.05). The PM subscale is correlated with BIS-11 attentional, motor impulsiveness dimensions (*p* < 0.05). The PM subscale is correlated with PCL-R interpersonal, lifestyle, and antisocial dimensions (*p* < 0.05). The IA subscale is not correlated with PCL-R. The Portuguese translated version of IPAS has adequate psychometric properties, allowing the measurement of impulsive and premeditated dimensions of aggression.

## Introduction

Aggression represents a public health issue. Having a negative impact on individuals and society (1), it can be defined as a “behavioral display in which physical force is used with the intent to harm or damage another individual or object” (2).

A clinical definition of aggressive behavior should consider biological, environmental, cultural, and social variables, and their interplay to act as predisposition or eliciting factors (3). The range of behaviors that can be classified as aggressive can vary from verbal aggression to homicide, and it is important to characterize the level of planning, the possible understanding of hypothetical consequences, the presence of frustrations, insults, interpersonal attack, threats, environmental stressors, and associated psychopathologies.

Empirical literature has proposed a dichotomous aggression classification, i.e., impulsive aggression (IA; reactive, affective, or non-planned), and premeditated aggression (PM; proactive, instrumental, predatory, or controlled) (4). The expression “IA” refers to uncontrolled aggressive outbursts that are out of proportion to the provoking event, while “PM” describes aggressive behaviors that are planned, controlled, and/or goal-oriented (5).

According to the aggression type, individuals may differ in social adjustment, criminal behavior, emotional function, cognitive performance, autonomic response, and treatment outcome (6, 7).

The IA tends to occur in the presence of triggering stimulus that is interpreted as threat or provocation (8). This type of aggression involves affective arousal, leading to a rapid and uncontrolled behavioral response. It has been correlated with information-processing/neurocognitive impairments, abusive home backgrounds, an angry/impulsive/anxious personality, and high psychological stress reactivity (9).

The PM, by contrast, tends to be a planned behavior that has a specific expected goal (8), and it can be explained as a learned behavior (10). It has been correlated with poor parental control, lack of affect, psychopathic personality, and low physiological arousal (9).

The classification of an aggressive act or a pattern of aggressive behavior allows treatment selection and violence risk management. The IA has a good response to pharmacologic treatment when mood stabilizers and/or antipsychotics are prescribed (11). In the case of PM, on the contrary, the response to pharmacological treatment is generally insufficient (12), and forensic/behavioral strategies are recommended (7). Furthermore, individuals with PM have a higher risk of violent criminal recidivism than those with IA (13).

There is a link between aggressive behavior and psychiatric disorders (14).

Individuals with depression, anxiety, psychosis, substance use disorders, hyperactive attention deficit disorder, and personality disorders present a higher frequency of aggressive acts (15).

Impulsive aggression has been linked to psychiatric diseases, such as anxiety, depression, personality disorders, and substance use disorders (16). PM has been associated with psychopathic personality traits (17).

In particular, antisocial personality disorder (ASPD) is characterized by significant irritability, agitation, impulsiveness, and hostility, and aggression represents one of its core symptoms (18). ASPD is highly prevalent among inmates. It is, thus, responsible for adverse events and elevated direct and indirect economic costs in the criminal justice system (19).

Antisocial personality disorder is the only acknowledged psychiatric disorder that confers an increased risk for both IA and PM (20–22), with several studies reporting psychopathy and psychopathic traits as risk factors for PM (23).

Psychopathic Checklist Revised (PCL-R) is the gold standard for psychopathy assessment and diagnosis, and separates psychopathic traits into four dimensions: (1) interpersonal, (2) affective, (3) lifestyle, and (4) antisocial (24).

Impulsivity has been related to aggression and is a symptom of ASPD (25). Impulsivity can be a necessary factor for aggression development (26). It can be defined “as a predisposition toward rapid, unplanned reactions to internal or external stimuli without regard to the negative consequences of these reactions to the impulsive individuals or to others” (27).

Impulsivity can be measured with Barratt Impulsiveness Scale, 11th version (BIS-11), a self-report psychometric instrument that separate impulsivity into three components: (1) acting on the spur of the moment (motor activation), (2) not focusing on the task at hand (attention), and (3) not planning and thinking carefully (lack of planning) (28). Individuals with scores higher than 72 are considered highly impulsive (29).

Patients with substance use disorders, depression, bipolar disorder, attention deficit/hyperactivity disorder (AD/HD), as well as suicide attempters and criminal offenders tend to have higher BIS-11 total scores (29).

Despite the evidence supporting the notion that persons who display an impulsive aggressive behavior are distinct from their counterparts who present a premeditated behavior, the clinical usefulness and generalization is questioned because most studies included violent incarcerated offenders, or psychiatric inpatients.

Within the incarcerated population, however, aggression is a relevant daily clinical concern. Therefore, the study of aggression in forensic psychiatric settings should be encouraged in order to find and promote the best clinical practices (30, 31).

It is important to use validated instruments in the evaluation of aggressive acts in prisoners in order to improve medical interventions (32). In Portugal, we do not have a validated aggression categorization instrument.

There are two psychometric instruments that allow aggression categorization: the impulsive–premeditated aggression scale (IPAS) and the reactive–proactive aggression questionnaire (2, 33, 34).

In the original validation study of IPAS, the sample comprised 93 physically aggressive men recruited in the community with a mean age of 35.9 years, whose principal aggression type was physical assault (*n* = 80). The aggression was measured through the Lifetime History of Aggression, Buss Perry Aggression Questionnaire, and the Aggression Interview. The principal component analysis identified two factors (IA and PM), which accounted for 16.56 and 14.03% of total variance, respectively. The internal consistency measured by Cronbach’s alpha was 0.77 for the IA factor and 0.82 for the PM factor. Sensitivity for the IA scale was 0.96 and specificity was 0.50. For the PM scale, sensitivity and specificity were 0.60 and 0.96, respectively (2).

All of the validation studies of IPAS reported identical results in principal component analysis, with two factors IA and PM. Internal consistency coefficients varied between 0.70 and 0.93 for IA and between 0.66 and 0.90 for PM (Table 1).

**Table 1:** Validation studies of Impulsive/Premeditated Aggression Scale (IPAS).

Reference	Sample			Mean age^a^	Principal aggression	Aggression measures	Internal consistency^b^		Total explained variance^c^	

	*n*	Type	Origin				IA^d^	PM^e^	IA	PM
Stanford et al. (2)	93 men	Physically aggressive men	Community	35.9	86% physical assault	LHAQ^f^, BPAQ^g^, AI^h^	0.77	0.82	16.56	14.03
Kockler et al. (35)	86 men	Prisoners	Inpatient Forensic hospital	38	83% convicted for violent crimes^i^, 37% non-guilty by reason of insanity	ns^j^	0.81	0.72	20	13
Conner et al. (36)	61 women, 60 men	Patients in treatment for opiate dependence	Outpatient Psychiatric Service	41.9	ns	LHAQ, BPAQ	0.74	0.75	ns	
Mathias et al. (37)	24 girls, 42 boys	Adolescent s with conduct disorder	Community	14.5	ns	LHAQ, BPAQ	0.82	0.78	18.5	15.6
Stanford et al. (38)	113 men	Men convicted of domestic violence	Community	36	Domestic violence	LHAQ	0.75	0.86	ns	
Haden et al. (39)	213 women, 127 men	Students	Community	19.06	ns	BPAQ	0.77	0.81	24	12
Kuyck et al. (40)	149 men, 70 women	Prisoners	Prison	50% between 25 and 39	75% convicted for violent crimes	ns	0.93	0.90	ns	
Chen et al. (41)	389 women, 262 men	Students	Community	26.1	ns	BPAQ	0.70	0.66	12.9	11.9
Romans et al. (42)	114 women, 49 men	Psychiatric patients	Outpatient Psychiatric Service	25.8	ns	OAS^k^	0.85	0.76	23.3	10.1

*^a^Years*.

*^b^Cronbach’s alpha*.

*^c^%*.

*^d^Impulsive aggression factor*.

*^e^Premeditated Aggression factor*.

*^f^Lifetime History of Aggression*.

*^g^Buss Perry Aggression Questionnaire*.

*^h^Aggression Interview*.

*^i^Aggravated assault, battery on a law officer, sexual assault, murder, armed robbery, attempted murder, and manslaughter*.

*^j^Not specified*.

*^k^Overt Aggression Scale*.

We intend to validate the Portuguese version of the IPAS in inmates and to determine its psychometric properties. Thus, we expect to replicate the purported two-factor model of IPAS; we predict that the subscales of the IPAS and the BIS-11 will be positively correlated; we predict that the PM subscales of the IPAS—unlike the IA ones—will be correlated with the PCL-R.

This study is a part of a larger study aiming to characterize biological and psychosocial factors that predict the aggression type in young adult male inmates.

## Materials and Methods

Our sample was collected at two penitentiary institutions in the North of Portugal. The research protocol was formally approved by the Ethics Committee of *Centro Hospitalar de São João*, Porto, Portugal (Document number 48.14), and by the hosting institution, the General Direction of Probation and Prison Services. Participation was voluntary and there was no reward for it. The participants were all Portuguese. Written informed consent was obtained after the procedures were explained to the participants, in accordance with the Declaration of Helsinki.

Participants were included if they were over 18 years old, had a personal history of aggression in the past 6 months, had been referred to the clinical services for aggressions toward other inmates, were able to read, and if they were capable of providing their written informed consent. Socio-demographic characteristics and forensic history were collected from interviews and clinical records. We applied three psychometric instruments: the IPAS, the BIS-11, and the PCL-R. This sample does not intend to represent all the Portuguese inmate population, as the methods involve a non-probabilistic sample approach.

### Transcultural Adaptation and Validation

In order to address the transcultural adaptation and validation of IPAS, an initial translation was carried out by a group of Portuguese experts. The back-translation of this first translated version was then performed by a native English-speaking expert. The first group analyzed the semantic and idiomatic correspondence of the translation and the back-translation, and performed a final synthesis translation (43).

### Psychometric Instruments

#### Impulsive–Premeditated Aggression Scale

The IPAS is a 30-item self-report questionnaire used to classify aggressive acts occurring over the previous 6 months. Items are scored within a five-point Likert scale (1 = Strongly Disagree to 5 = Strongly Agree). The scale differentiates two factors: IA and PM, with the weighted score allowing for the categorization of the type of aggression. The individual’s level of IA and PM was obtained through the sum of 20 of the 30 items of the IPAS: the IA items (eight items: 3, 5, 7, 8, 9, 21, 24, and 26) and the PM items (12 items: 1, 2, 6, 10, 11, 12, 14, 16, 17, 20, 29, and 30) (2).

#### Barratt Impulsiveness Scale-11

To determine the convergent validity of the IA and the PA subscales of the IPAS, we have utilized BIS-11. The BIS-11 is a self-report questionnaire for assessing general impulsiveness. The current scale version contains 30 items that are coded from 1 (rarely/never) to 4 (almost always/always). The level of impulsiveness is calculated by summing up the scores for each item. All items were defined as identifying impulsiveness within the structure of related personality traits. The factor analysis revealed three components as follows: “attentional impulsiveness,” “motor impulsiveness,” and “non-planning impulsiveness.” Instead of a unique total score, the study of the individual contribution of each component is recommended. Findings report an acceptable/high internal consistency (Cronbach’s alpha 0.79–0.82) of the scale when applied to forensic samples (28). The structural properties of the BIS-11 were replicated in Portuguese speaking samples (44).

#### Psychopathy Checklist Revised

To determine the divergent validity of the IA and the PA subscales of the IPAS, we have applied the PCL-R. The PCL-R is the gold standard measure of psychopathy, gathering information from records and a semi-structured interview (24, 25). The 20 items are scored as absent (0), present to some degree (1), or fully present (2), having a maximum total score of 40 points. This instrument may be considered a four-factor model comprising interpersonal, affective, lifestyle, and antisocial dimensions (45). The interpersonal and affective dimensions jointly serve as second-order factors representing the core traits of the psychopathic personality. Lifestyle and antisocial facets form a superordinate factor of social deviance (46). The two second-order factors (Factor 1 and Factor 2) are concomitant with the original factor structure reported for the first edition of the PCL-R (47). The structural properties of the PCL-R were replicated in Portuguese samples for the standard protocol, including record review and structured interview (48). The PCL-R was applied by an experienced psychiatrist (Jacinto Costa Azevedo).

### Statistical Analyses

Statistical analyses were conducted following the methodology used in the previous IPAS validation studies in adults. The principal component analysis was conducted with no assumptions regarding the number of potential factors. Lautenschlager’s (49) tables were used to determine the threshold for significant factors (minimum eigenvalue of 2.0) and item factor loadings (minimum eigenvalue of 0.40). In order to assess factor structure and maximize the reliability of factor interpretation, an Oblimin rotation was used so as to obtain an oblique factor solution of the original factors (50). Internal consistency was measured using Cronbach’s alpha (51). Convergent and divergent validity of the IPAS was tested by examining the Pearson’s product-moment correlations with the standardized measures of impulsivity and personality (BIS-11 and PCL-R). Confirmatory factorial analysis was tested by calculating goodness-of-fit indice, robust method (52).

The analyses were carried out using IBM SPSS Statistics for Windows, Version 22.0 (Armonk, NY, USA: IBM Corp.), and with the Structural Equation Program, EQS version 6.1 (53).

## Results

### Sample Description

The sample comprised 240 inmates with a mean (±SD) age of 35.4 ± 8.4 years; a total of 67.1% (*n* = 161) of the participants were single, and 58.3% did not have any children (*n* = 140). The mean education level of the sample was 6.8 ± 3.2 years. The mean time in prison (time spent behind bars at the time of assessment) of the sample was 109.3 ± 70.6 months. A total of 45.8% (*n* = 110) of the inmates had been convicted of violent crimes (physical assault, murder, attempted murder). Validation procedures were performed in the total sample (*n* = 240).

### Principal Component Analysis

We have performed a principal component analysis exploring the original 30 items. First, we inspected the Kaiser–Meyer–Olkin Measure of Sampling Adequacy (KMO = 0.87) and reached the conclusion that it was an appropriate value. Second, we achieved a statistically significant Bartlett’s Test of Sphericity (*p* < 0.01). Third, we extracted two factors (with an oblimin rotation) with eigenvalues of 7.19 (Factor 1, IA) and 3.29 (Factor 2, PM) accounting for 34.27 and 15.65% of total variance, respectively. The IA factor was composed of ten items: 30, 27, 22, 9, 24, 15, 26, 4, 7, and 13. The PM factor was composed of eleven items: 6, 14, 29, 28, 2, 23, 12, 16, 20, 10, and 1. The items 3, 5, 8, 11, 17, 18, 19, 21, and 25 were excluded from the analysis as they exhibited component loadings inferior to 0.40 (Table 2).

**Table 2:** Factor loadings of Impulsive/Premeditated Aggression Scale (IPAS).

IPAS Items	Impulsive	Premeditated
30. Anything could have set me off prior to the incidents	0.63	–
27. I was in a bad mood the day of the incident	0.66	–
22. I was confused during the acts	0.79	–
9. I feel I lost control of my temper during the acts	0.80	–
24. My behavior was too extreme for the level of provocation	0.72	–
15. I became agitated or emotionally upset prior to the acts	0.72	–
26. I consider the acts to have been impulsive	0.66	–
4. I typically felt guilty after the aggressive acts	0.73	–
7. I usually can’t recall the details of the incidents well	0.50	–
13. I feel some of the incidents went too far	0.72	–
6. I feel my actions were necessary to get what I wanted	–	0.58
14. I think the other person deserved what happened to them during some of the incidents	–	0.65
29. I am glad some of the incidents occurred	–	0.64
28. The acts were a “release” and I felt better afterward	–	0.75
2. I felt my outbursts were justified	–	0.70
23. Prior to the incidents I knew an altercation was going to occur	–	0.55
12. I wanted some of the incidents to occur	–	0.80
16. The acts led to power over others or improved social status for me	–	0.72
20. Some of the acts were attempts at revenge	–	0.75
10. Sometimes I purposely delayed the acts until a later time	–	0.63
1. I planned when and where my anger was expressed	–	0.64
Eigenvalues	7.19	3.29
Variance (%)	34.27	15.65
Cronbach’s alpha	0.89	0.88

### Confirmatory Factorial Analysis

To test the hypothesis of two dimensions, we use the confirmatory factor analysis, to test the goodness-of-fit indice, robust method. Results showed a comparative fit index of 0.90, and a root mean square error of approximation (RMSEA) of 0.06, 90% confidence interval (0.05, 0.08).

### Internal Consistency

The internal consistency test of the dimensions obtained in the factor analysis with the present sample revealed a good Cronbach’s alpha value for the IA factor (0.89), and a good Cronbach’s alpha value for the PM factor (0.88) (Table 2).

Reliability was also tested regarding the original version of the IPAS subscales. Cronbach’s alpha for the IA original subscale was acceptable (0.78), and it was good for the PM subscale (0.85).

### Convergent and Divergent Validity

We have studied the convergence between the IPAS and the BIS-11. The statistically significant correlations between the two scales (IPAS and BIS-11) and between its dimensions are detailed in Table 3, showing correlations with attentional, motor, and non-planning dimensions (2, 5, 28). Correlations were statistically significant, suggesting the existence of convergence between the two scales.

**Table 3:** Pearson correlations coefficients between Impulsive/Premeditated Aggression Scale (IPAS), Barratt Impulsiveness Scale, 11th version (BIS-11), and Psychopathic Checklist Revised (PCL-R).

		IPAS subscales	

Measures	Subscales	IA	PM
BIS-11	Total score	0.21**	0.20**
	Attentional	0.14*	0.18*
	Motor	0.26**	0.27**
	Non-planning	0.15*	0.06
PCL-R	Total score	0.01	0.29**
	Interpersonal	−0.14	0.24*
	Affective	−0.14	0.16*
	Lifestyle	−0.07	0.22*
	Antisocial	−0.07	0.21*

*The study used 240 participants for correlations between IPAS and BIS-11; and a subsample of 134 participants for correlations between IPAS and PCL-R*.

*IA, impulsive aggression; PM, premeditated aggression*.

*p < 0.05; **p < 0.01.

We have studied divergent validity through the analysis of correlations between IPAS and PCL-R in a subsample of 134 inmates. The statistically significant correlations between the two scales (IPAS and PCL-R) and between its dimensions are detailed in Table 3. The IA subscale is not correlated with PCL-R. The PM subscale is correlated with PCL-R interpersonal, lifestyle, and antisocial dimensions (*p* < 0.05).

## Discussion

According to AERA-APA-NCME (54), theoretical and empirical pieces of evidence that support the interpretations of test scores are fundamental so as to indicate the degree to which scores capture important aspects of the construct, as well as its relevance to the proposed use. The present results show appropriate validity for the Portuguese version of the IPAS.

The items of the instrument were adapted according to the original version (2). In our sample, IPAS was able to demonstrate the presence of two factors: impulsive and PM.

All the authors who studied the validity of IPAS were able to reduce the variability of the scale to two factors: IA and PM factors. This is in line with the literature on human aggression which argues that aggression is not a single construct and should be understood as being composed of two dimensions or categorized into two different types: impulsive or PM.

In the analysis of the explained variance described by other authors, we have observed that the IA factor can explain between 12.9% (41) and 24% (39) of the total IPAS variance; and that the PM factor can account for between 10.3% (42) and 15.6% (37) of the total IPAS variance. We have obtained higher values of explained variance (34.3% for the IA factor and 15.7% for the PM factor). In the analysis of the IPAS internal consistency described by other authors, we have observed that it varies between 0.70 (41) and 0.93 (40) for the IA factor; and between 0.66 (41) and 0.90 (40) for the PM factor. Our values of internal consistency and variance can be explained by the homogeneity of the sample.

According to Bentler and Douglas (52), when the goodness-of-fit and adjusted goodness-of-fit indexes are higher than 0.90, the analyses indicate adequate fit of the models. Also, according to Bentler and Douglas, when the RMSEA is less than. 0.10, the analysis indicates adequate fit of the models.

In our study, we have assessed the convergent validity through the analysis of Pearson product-moment correlations coefficients between IPAS and BIS-11. We had significant correlations between the IA subscale and the BIS-11 motor activation, attention, and non-planning dimensions. We had significant correlations between the PM subscale and the BIS-11 motor activation, attention dimensions, but not for non-planning dimension.

We have found a slightly higher correlation between the total score of BIS-11 and IA than with the PM subscale of IPAS. Our results were line with Mathias and colleagues who reported significant correlations between the IPAS subscales and BIS-11’s total score (correlations coefficients of 0.39 for AI and 0.26 for PM) (37).

However, Stanford and colleagues have reported higher significant correlations between PM and the total score of BIS-11 (correlation coefficients of 0.21 for AI and 0.38 for PM) (2).

Our data reveal significant correlations between the three dimensions of impulsivity measured by BIS-11and IA subscale of IPAS. Similar results were reported by Chen and colleagues that obtained significant values for correlations between IA and the three dimensions of BIS-11 (correlation coefficients of 0.15 for attentional impulsiveness, 0.21 for motor impulsiveness, and 0.12 for non-planning impulsiveness and AI) (41).

In the case of PM subscale of IPAS, we did not observe significant correlations with the non-planning dimension of BIS-11. The same was reported by Chen and colleagues, who only reported a significant correlation of 0.19 between PM and attentional impulsiveness (41).

We can explain these results in three possible ways. First, it is assumed that impulsivity is related aggression, not doing any discrimination between dimensions and type of aggression (33). These results are in accordance with the hypothesis that impulsivity is related and can be a predisposing factor for aggression (26). On the other hand, we should note that there are impulsive individuals who are not aggressive, and therefore, impulsivity is not the only factor necessary for the development of aggressiveness (55). Second, since our sample is composed of inmates, we can assume a high prevalence of individuals with substance use disorders and ASPD (56). In these types of pathology, there are a high expression of impulsivity (57, 58).

Thus, we can explain the correlations between subscales of IPAS and BIS-11 subscales because they are individuals with greater impulsivity conferred by underlying psychopathology. The third possible explanation, related to the non-planning dimension of impulsivity, is perhaps those impulsive individuals who maintain planning ability are those who can develop and learn premeditated aggressive behavior. These findings support the definition of PM in which individuals with PM plan their action and, thus, have fewer impairments in action planning impulsivity dimension (9). The non-planning dimension of impulsivity is related to working memory (59) and executive functions, namely with the subscale of strategic planning of the executive function index (60).

These results support the need for use of appropriated psychometric scales for the evaluation of aggression, in order to go beyond the assumption that individuals with higher levels of impulsivity are more likely to display externalizing behavior and thus having a greater probability of showing aggressive behavior.

This was the rationale for the use of BIS-11 for the evaluation of convergent validity with IPAS.

We have evaluated the divergent validity by analyzing correlations between AI and PM factors and PCL-R. As a result, we have obtained significant correlations for PM and PCL-R interpersonal, lifestyle, and antisocial dimensions, and not significant correlations for AI and PCL-R. Stanford and colleagues, in 2008, evaluated correlations between Psychopathic Personality Inventory (PPI) and the AI and PM factors, having obtained significantly higher values of PPI in the individuals categorized as premeditated aggressors, that is, having higher PM scores (38).

These data are in agreement with the literature on psychopathy and the type of aggressiveness externalized by individuals with higher expression of psychopathic personality traits. Psychopathy seems to be a risk factor for PM, mainly in forensic samples (23).

Regarding the generalization of results, some limitations should be considered. Although we have worked with a large sample, it is exclusively composed by men, therefore we do not know if aggressive women will behave in the same manner. The participants are subject to long sentences, making it difficult to generalize the results so as to include inmates with shorter sentences. Also, participation in this kind of research in a forensic facility context can change the way inmates respond to the IPAS. We must take into consideration that the legal circumstances of participants can modify the type of answers. This sample was obtained in a prison for convicted individuals, in Northern Portugal. We do not know whether the scale psychometric characteristics are the same in other forensic samples. In future research, it may be useful to consider tools that can externally quantify the individual acts of aggression, such as the Modified-Overt Aggression Scale.

## Ethics Statement

The research protocol was formally approved by the Ethics Committee of Centro Hospitalar de São João, Porto, Portugal (Document number 48.14). Written informed consent was obtained after the procedures had been explained to participants, in accordance with the Declaration of Helsinki.

## Author Contributions

JA and MF-B contributed to the conception of the study. The data analysis was carried out by JA and JP. All authors contributed to the interpretation of the data. JA and MF-B wrote the manuscript. All authors revised the content critically and approved the final version.

## Conflict of Interest Statement

The authors report no conflicts of interest. The authors alone are responsible for the content and writing of the paper.
